# Genome Sequence of *Salmonella enterica* subsp. *enterica* Serovar Typhi Isolate PM016/13 from Untreated Well Water Associated with a Typhoid Outbreak in Pasir Mas, Kelantan, Malaysia

**DOI:** 10.1128/genomeA.01261-15

**Published:** 2015-11-12

**Authors:** Salwani Muhamad Harish, Kee-Shin Sim, Nazalan Najimudin, Ismail Aziah

**Affiliations:** a Institute for Research in Molecular Medicine (INFORMM), Health Campus, Universiti Sains Malaysia, Kubang Kerian, Kelantan, Malaysia; b School of Biological Science, Main Campus, Universiti Sains Malaysia, Pulau Pinang, Malaysia

## Abstract

*Salmonella enterica* subsp. *enterica* serovar Typhi is a human-restricted pathogen that causes typhoid fever. Even though it is a human-restricted pathogen, the bacterium is also isolated from environments such as groundwater and pond water. Here, we describe the genome sequence of the *Salmonella enterica* subsp. *enterica* serovar Typhi PM016/13 which was isolated from well water during a typhoid outbreak in Kelantan, Malaysia, in 2013.

## GENOME ANNOUNCEMENT

Typhoid fever is a severe infection of the reticuloendothelial system (1). It is endemic in some countries where outbreaks usually occur due to unhygienic conditions and practices (2). It is estimated worldwide that at least 16 million cases and 500,000 deaths occur each year because of typhoid fever (3).

Kelantan has always been recorded as having a higher incidence of typhoid compared to other states of Malaysia (4, 5). In 2013, an outbreak was reported in Pasir Mas, Kelantan, and from the outbreak, a total of eight *Salmonella enterica* serovar Typhi isolates were obtained. These isolates were confirmed using culture method, serological test and polymerase chain reaction (PCR) (6). Seven isolates were from human stool samples while one isolate was obtained from well water samples. In this report, we announce the availability of a fully closed genome sequence of *Salmonella* Typhi PM016/13.

The genomic DNA of PM016/13 was extracted from overnight cultures using a genomic DNA buffer set and genomic tip 100/G (Qiagen, Inc., Valencia, CA) and sequenced using the Pacific Biosciences (PacBio) RS sequencing platform. A 10-kb library was prepared and sequenced using C_2_ chemistry on 3 single molecule real-time (SMRT) cells with a 180-min collection protocol on the PacBio RS. The 10-kb continuous-long-read (CLR) was *de novo* assembled using the PacBio hierarchical genome assembly process (HGAP)/Quiver software package, followed by Minimus 2, and were polished with Quiver. A single contig of 4,793,553 bp was generated from the assembly with 52% G+C content. The genome did not harbor any plasmid. The gene prediction was performed using Glimmer3 (7) and functional annotation performed by Blast2GO (A. Conesa et al., unpublished data) predicted 4,937 genes for protein-coding (CDSs) with an average length of 767 bp, 22 rRNA genes predicted using RNAmmer (8), and 80 tRNA genes predicted by ARAGORN (9).

The genome encodes a type III secretion system as reported previously in CT18 and Ty2 (10). The genome also coded for proteins such as the oxygen regulated invasion protein OrgA and OrgB which are reported to be within *Salmonella enterica* serovar Typhimurium pathogenicity island I (11, 12). It is also found that the genome encodes *Salmonella* invasion protein (Sip) D, which enables the organism to induce apoptosis in macrophages (10). Multidrug resistance proteins B and D were located within the genome as also reported in *Salmonella enterica* serovar Typhi CT18 and Ty2 (13). Several pathogenicity islands and hypothetical proteins were also identified in the genome sequence.

The genome of *Salmonella enterica* serovar Typhi is genetically diverse among human isolates (2, 4, 14–16). Thus, the availability of a complete genome of a *Salmonella enterica* serovar isolated from the environment can be used to improve the understanding of its genome diversity and persistence for adaptation that allows them to survive.

### Nucleotide sequence accession number.

This genome project has been deposited in GenBank under accession number CP012091.
